# The critical role of peroxiredoxin-2 in colon cancer stem cells

**DOI:** 10.18632/aging.202784

**Published:** 2021-03-26

**Authors:** Linglong Peng, Yongfu Xiong, Rong Wang, Ling Xiang, He Zhou, Zhongxue Fu

**Affiliations:** 1 Department of Gastrointestinal Surgery, The Second Affiliated Hospital of Chongqing Medical University, Chongqing 400014, China; 2 Department of Hepatobiliary Surgery, The Affiliated Hospital of North Sichuan Medical College, Sichuan 637000, China; 3 Department of Oncology, The Cancer Center of the Fifth Affiliated Hospital of Sun Yat-sen University, Zhuhai 519000, China; 4 Department of Clinical Nutrition, The Second Affiliated Hospital of Chongqing Medical University, Chongqing 400014, China; 5 Department of Gastrointestinal Surgery, The First Affiliated Hospital of Chongqing Medical University, Chongqing 400014, China

**Keywords:** colon cancer, colon cancer stem cells, peroxiredoxin-2, metastasis, chemoresistance

## Abstract

Colon cancer stem cells (CCSCs) play an important role in facilitating colon cancer occurrence, metastasis and drug resistance. The results of our previous studies confirmed that the well-studied antioxidant gene peroxiredoxin-2 (PRDX2) promotes colon cancer progression. However, the underlying function and mechanisms associated with PRDX2 remodeling in the context of CCSCs have remained poorly studied. In our present study, we demonstrated that PRDX2 is highly expressed in CD133/CD44-positive colon cancer tissues and spheroid CD133+CD44+ CCSCs. PRDX2 overexpression was shown to be closely correlated with CD133+CD44+ CCSCs in colon cancer. Furthermore, PRDX2 depletion markedly suppressed CD133+CD44+ CCSC stemness maintenance, tumor initiation, migration and invasion and liver metastasis. Furthermore, the expression of various EMT markers and Wnt/β-catenin signaling proteins was altered after PRDX2 inhibition. In addition, PRDX2 knockdown led to increased ROS production in CD133+CD44+ CCSCs, sensitizing CCSCs to oxidative stress and chemotherapy. These results suggest that PRDX2 could be a possible therapeutic target in CCSCs.

## INTRODUCTION

Despite the continued development of medical technology, the incidence and mortality of colon cancer has not been significantly reduced worldwide [1]. Most colon cancer deaths occur when the cancer has metastasized to other tissues, most commonly the liver [2]. Recently, the cancer stem cell (CSC) model has been promoted as a cornerstone of the development of cancers and is increasingly viewed as the cause of cancer recurrence and spread [3, 4]. Bonnet et al. first described this model in human acute myeloid leukemia and then confirmed it in many solid cancers, including colon cancer [5–7]. The CSC model suggests that only a small number of tumor cells are capable of self-renewal, have an enhanced potential to drive tumor metastasis and exhibit increased resistant to therapy [8].

CSCs have been identified through their expression of putative cell surface markers [9]. Currently, colon cancer stem cell (CCSC) populations have been isolated and identified by several markers, such as CD133, CD44, CD166, Lgr5, EpCAM, ALDH1 and β-catenin [10–17]. Using CD133 alone or in combination with other markers, many types of CSCs have been enriched, including pancreatic, liver, brain, colon, and kidney cancers [18]. CD44, an important transcriptional regulator of the WNT/β-catenin pathway, is another commonly used marker for isolating CCSCs [13]. CD44 is involved in regulating a variety of biological behaviors, especially cell-cell interactions, stemness, and tumor metastasis and progression [19, 20]. CD44 was shown to be extensively expressed in the colonic stem cell niche located in the crypt base of normal or tumor colonic cells [21]. Based on the characteristics of CD133 and CD44, several researchers aimed to screen an ideal CCSC population. Botchkina et al. demonstrated that CD133^high^CD44^high^ cells isolated from HCT116, HT29, and DLD1 cell lines are undifferentiated with epithelial lineage differentiation and exhibit extensive self-renewal abilities *in vitro* [22]. In addition, several studies demonstrated that CD133^+^CD44^+^ cells exhibited much higher invasion and migratory capacities *in vitro* than a non-CCSC subset and drove tumor metastasis formation *in vivo* [23, 24]. Moreover, both CD133- and CD44-positive cells are more resistant to anticancer therapies and are closely associated with tumor progression and poor prognosis [9, 25, 26]. Taken together, this suggest that CD133^+^CD44^+^ cells may be an ideal candidate for the isolation and further characterization of CCSCs and serve a useful therapeutic target for the elimination of CCSCs.

Peroxiredoxin-2 (PRDX2) is a typical 2-Cys thioredoxin peroxidase known to catalyze peroxide reduction to balance cellular levels of hydrogen peroxide (H_2_O_2_) [27, 28]. Previous studies detected high PRDX2 levels in various types of malignant epithelial tumors including colorectal cancer, and PRDX2 was shown to promote tumor formation by regulating various cancer-related cellular signaling pathways [29–35]. In addition, PRDX2 is a diagnostic marker in squamous cervical cancer, a predictive indicator for the induction chemotherapy response in osteosarcoma, and a prognostic biomarker in ovarian cancer and colorectal cancer [36–39]. The results of these studies suggest that PRDX2 plays a crucial role in the occurrence and development of human cancer.

Currently, growing evidence suggests the ineffectiveness of anticancer therapy is attributable to the existence of CSCs, which are responsible for the sustainment, recurrence, metastasis and resistance of tumors to treatment [4, 6]. CSCs are biologically distinct from differentiated cancerous cells. Although some anticancer therapies have effectively eliminated bulk tumor cells, they have been unable to successfully clear CSCs [7]. Thus, effective anticancer treatment must be specifically targeted toward CSCs, not only bulk tumor cells. Since CCSC-enriched CD133^+^CD44^+^ cells are more resistant to conventional therapies than the other differentiated tumor cells, and based on our previous results that PRDX2 is essential for maintaining the colon cancer stem cell-like phenotype [40], the significance of PRDX2 in CD133^+^CD44^+^ CCSCs was investigated in the present study.

## RESULTS

### PRDX2 expression is closely associated with CD133^+^CD44^+^ CCSCs in colon cancer

**Figure 1:**
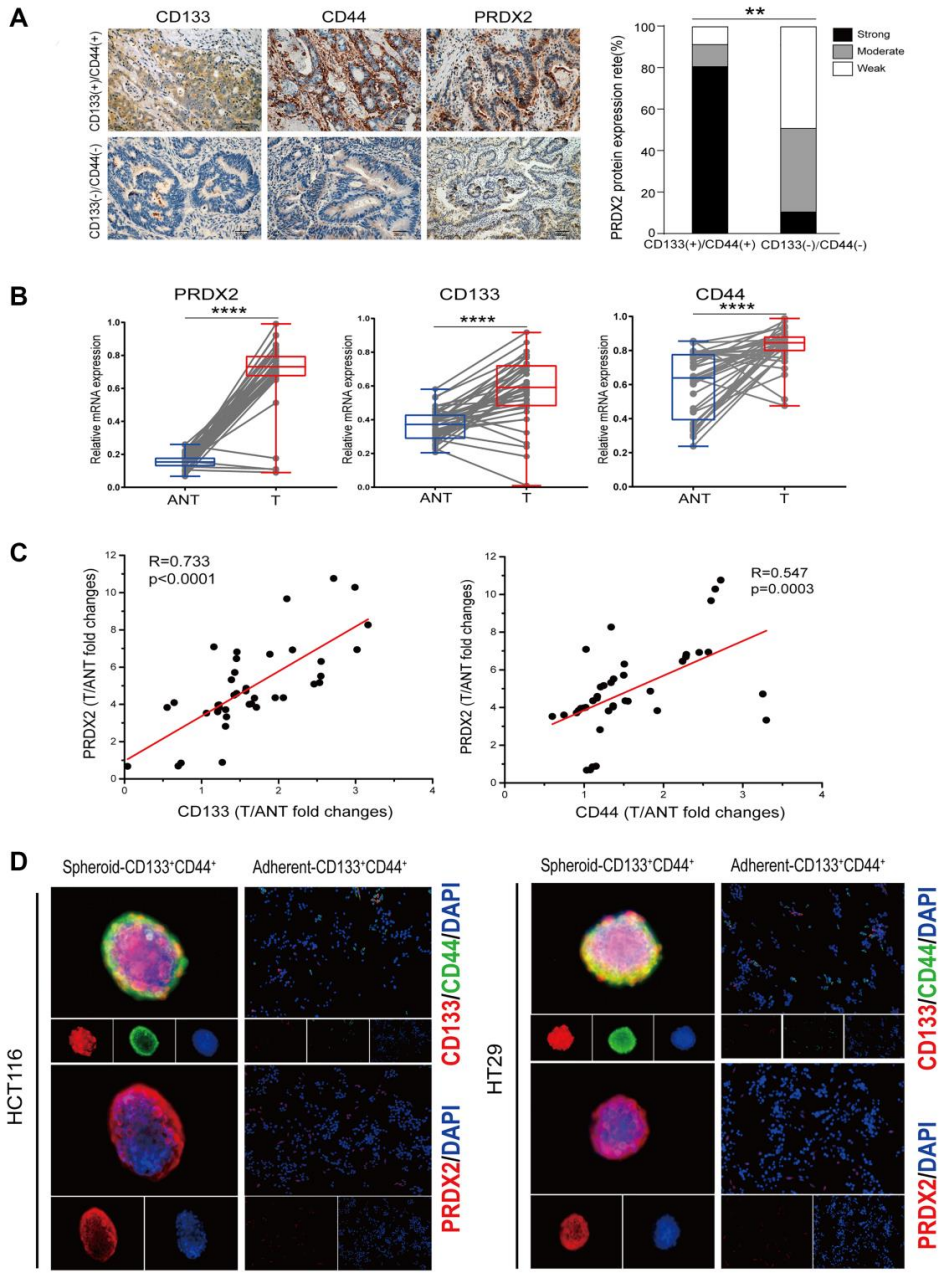
**PRDX2 expression is closely associated with CD133^+^CD44^+^ CCSCs in colon cancer.** (**A**) Left panel: Representative IHC images of CD133(+)/CD44(+) colon cancer tissues showing strong PRDX2 expression compared to CD133(-)/CD44(-) colon cancer tissues with weak PRDX2 expression. Right panel: Comparison of the proportions of PRDX2 expression in CD133(+)/CD44(+) colon cancer tissues (*n* = 9) and CD133(-)/CD44(-) colon cancer tissues (*n* = 10). Statistical analysis: Fisher’s exact test, ^**^p < 0.01. (**B**) Quantitation of PRDX2, CD44 and CD133 mRNA expression in 40 paired human colon tumor tissues (T) and matched adjacent noncancerous tissues (ANT) by real-time reverse transcription PCR analysis. Relative values were normalized to GAPDH. Statistical analysis: Paired *t*-test, ^****^p < 0.0001. (**C**) PRDX2 levels are correlated with CD133 and CD44 expression at the mRNA level. The data are presented as the fold changes in cancer specimens compared to matched adjacent noncancerous tissues. Statistical analysis: Nonparametric Spearman correlation analysis (*R* value) was performed. (**D**) Immunofluorescence analysis of PRDX2 expression and the coexpression of CD133 and CD44 in spheroid CD133^+^CD44^+^ CCSCs and adherent CD133^-^CD44^-^ cells isolated from HCT116 and HT29 cell lines, respectively.

Our previous study demonstrated that PRDX2 is overexpressed in colon cancer tissues compared to adjacent noncancerous tissues [29]. In the present study, to investigate the relationship between PRDX2 expression and CD133^+^CD44^+^ CCSCs, immunohistochemical (IHC) staining was conducted to determine the expression of PRDX2 in CD133(+)/CD44(+) and CD133(-)/CD44(-) colon cancer patients. The protein expression of PRDX2 was remarkably higher and frequently upregulated in CD133(+)/CD44(+) tumor tissues compared to CD133 (-)/CD44(-) tumor tissues (Figure 1A). In addition, we further analyzed whether PRDX2 transcriptional levels correlate with CD133 and CD44 expression in 40 pairs of human colon carcinomas with matched adjacent noncancerous tissues (ANTs). As shown in Figure 1B, the mRNA expression of PRDX2, CD133 and CD44 in tumor tissues was significantly upregulated compared to that observed in ANTs tissues. Then, the expression in relationships was analyzed, which demonstrated that the mRNA levels of PRDX2 were positively associated with those of CD133 and CD44 (Figure 1C). Furthermore, PRDX2 expression in spheroid CD133^+^CD44^+^ CCSCs and adherent CD133^-^CD44^-^ cells was also investigated by immunofluorescence staining. Notably, spheroid CD133^+^CD44^+^ CCSCs not only coexpressed CD133 and CD44 but also expressed PRDX2 enrichment (Figure 1D). In contrast, the adherent CD133^-^CD44^-^ cells showed minimal CD133 and CD44 expression, and only scattered PRDX2 was detected in these cells (Figure 1D). Taken together, these results indicate that PRDX2 expression is closely associated with CD133^+^CD44^+^ CCSCs in colon cancer.

### PRDX2 knockdown inhibits the self-renewal of CCSCs

**Figure 2:**
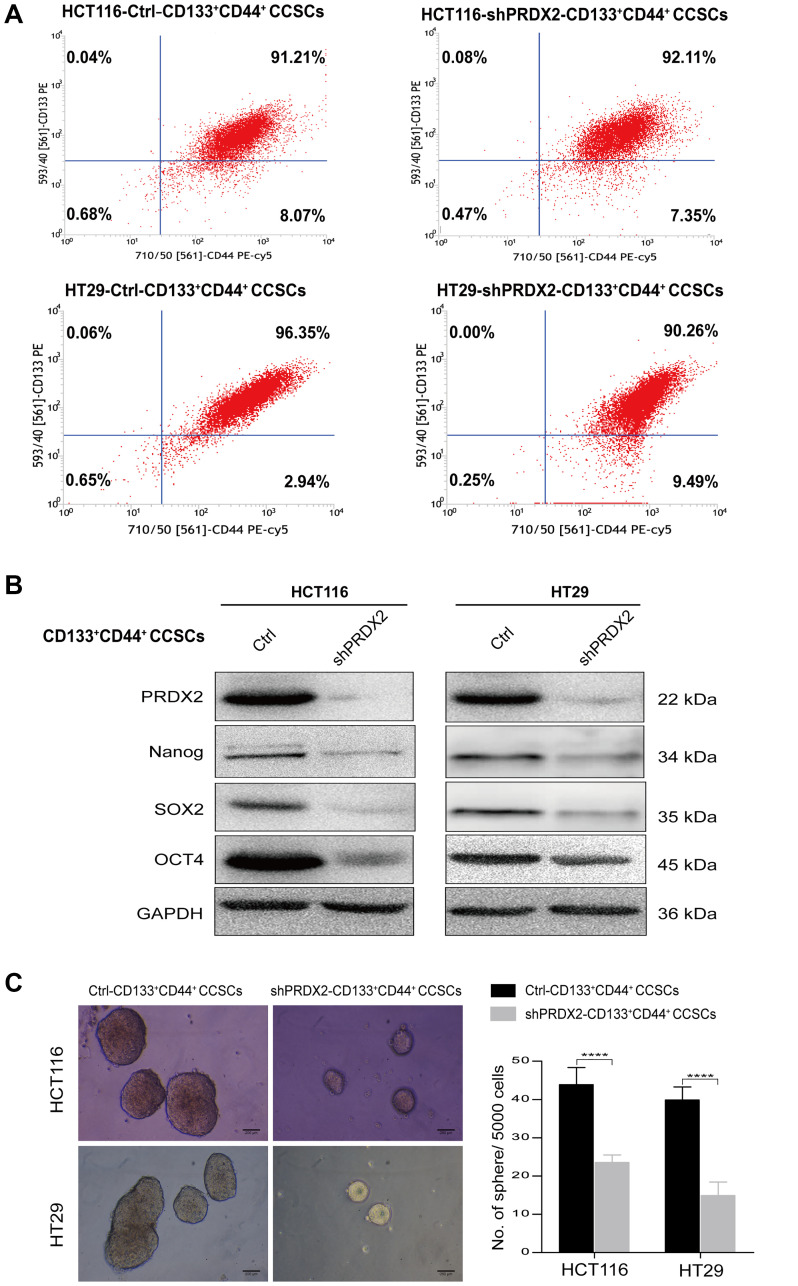
**PRDX2 knockdown inhibits the self-renewal of CCSCs.** (**A**) HCT116 and HT29 cell lines were first transfected with a lentiviral vector-mediated specific shRNA (shPRDX2) to silence PRDX2 or with a negative control lentivirus vector. Then, shPRDX2-CD133^+^CD44^+^ and control-CD133^+^CD44^+^ CCSCs were isolated from the transfected HCT116 and HT29 cells by magnetic bead sorting, which resulted in a considerable enrichment of CD133^+^CD44^+^ CCSCs (regular purity > 90%), as identified by flow cytometry with a PE-labeled anti-CD133 antibody and a PE-Cy5-labeled anti-CD44 antibody. (**B**) Western blot analysis of PRDX2 expression and the protein expression levels of stemness-related genes such as Nanog and Sox2 in CD133^+^CD44^+^ CCSCs generated from HCT116/HT29 control or shPRDX2 cells. GAPDH was used as the loading control. (**C**) Left panel: Representative image of spheres formed from CD133^+^CD44^+^ CCSCs derived from HCT116/HT29 control or shPRDX2 cells. Right panel: The number of cell spheres per 5000 cells was counted. The data are presented as the means ± SD of sphere numbers from three independent experiments performed in triplicate. Statistical analysis: Student’s *t*-test, ^****^p < 0.0001.

To investigate the significance of PRDX2 in CD133^+^CD44^+^ CCSCs, we first generated HCT116 and HT29 cells exhibiting stable PRDX2 knockdown (shPRDX2) via lentiviral vector-mediated specific shRNA delivery, and a nontarget negative control lentivirus vector was also transduced into these cells to control for the impact of the lentiviral vector. Next, shPRDX2-CD133^+^CD44^+^ and control-CD133^+^CD44^+^ CCSCs were isolated from these transfected cell lines by magnetic bead sorting, which led to a considerable enrichment of CD133^+^CD44^+^ CCSCs (regular purity > 90%) (Figure 2A). Using these procedures, we successfully silenced PRDX2 expression in CD133^+^CD44^+^ CCSCs from both HCT116 and HT29 cell lines (Figure 2A, Figure 2B and Supplementary Figure 1). Using these CCSCs, the stemness-related proteins were subsequently detected. CD133^+^CD44^+^ CCSCs derived from PRDX2 knockdown cells showed decreased expression of stemness-related proteins, such as Nanog, Sox2 and Oct4 compared to cells transfected with the scramble control (Figure 2B). In addition, in a sphere formation assay, PRDX2 knockdown significantly suppressed the ability of CD133^+^CD44^+^ CCSCs to form spheres *in vitro* (Figure 2C).

### PRDX2 knockdown suppresses the tumorigenic capacity of CCSCs

**Figure 3:**
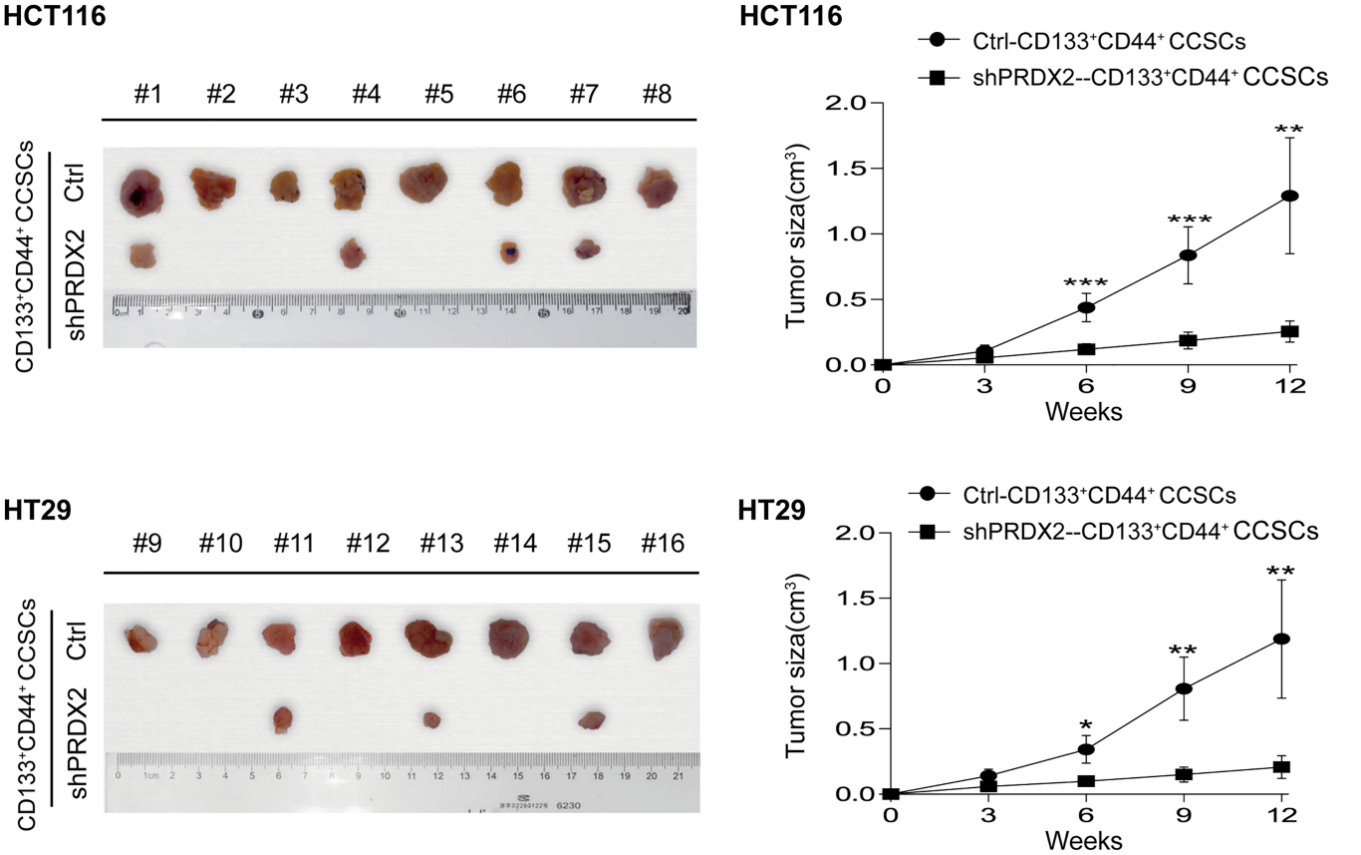
**PRDX2 knockdown suppresses the tumorigenic capacity of CCSCs.** Left panel: A total of 1 × 10^4^ cells dissociated from HCT116/HT29-control or -shPRDX2 CD133^+^CD44^+^ CCSCs were subcutaneously injected into the flanks of BALB/c nude mice (*n* = 8 per group). The incidence and size of subcutaneous tumors were counted and measured. Right panel: The growth of subcutaneous tumors was monitored. The image shows the growth curve of subcutaneous tumors of different cell populations at different time points. The tumor volume data are presented as the means ± SD. Statistical analysis: Student’s *t*-test, ^*^p < 0.05, ^**^p < 0.01, and ^***^p < 0.001 vs. shPRDX2-CD133^+^CD44^+^ cells.

To further characterize the role of PRDX2 in CD133^+^CD44^+^ CCSC tumorigenicity, a subcutaneous xenotransplant tumor models were established. Our results demonstrated that compared to the control CD133^+^CD44^+^ CCSCs, colon cancer cells from PRDX2 knockdown CD133^+^CD44^+^ CCSCs exhibited reduced tumor incidence, tumor size and tumor growth *in vivo* (Figure 3), suggesting that PRDX2 knockdown significantly suppresses the tumorigenic capacity of CD133^+^CD44^+^ CCSCs *in vivo*.

### PRDX2 depletion decreases the migration and invasion capacities of CCSCs

**Figure 4:**
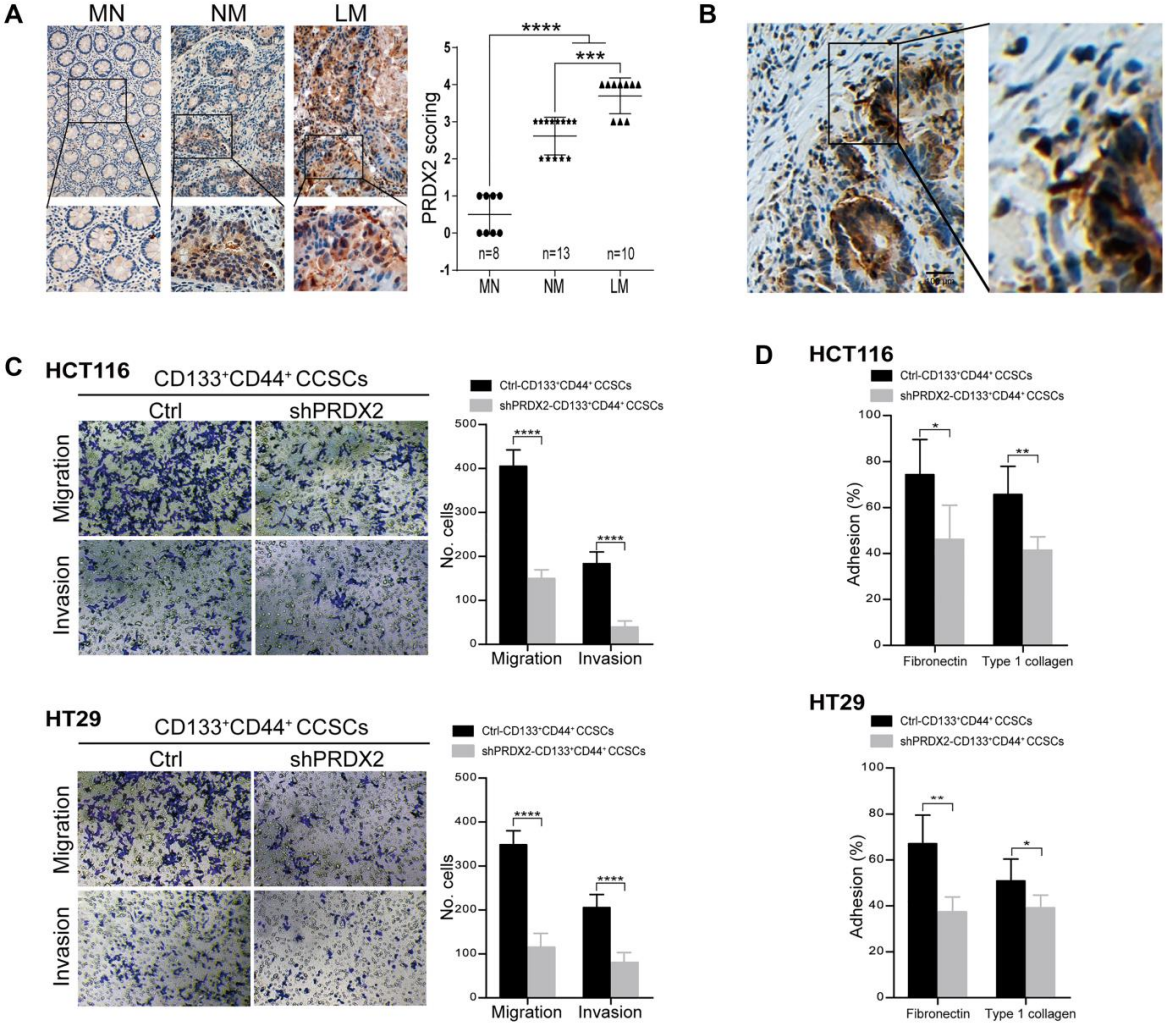
**PRDX2 depletion decreases the migration and invasion capacities of CCSCs.** (**A**) Left panel: Representative IHC staining for PRDX2 expression in matched normal (MN) colon tissues, nonmetastatic (NM) colon cancer tissues and colon cancer tissues with live metastasis (LM). The corresponding high-magnification images are also shown. Right panel: The PRDX2 expression intensity scores for 25 patient tissues, including MN (*n* = 8), NM (*n* = 13), and LM (*n* = 10). Statistical analysis: Fisher’s exact test, ^***^p < 0.001, and ^***^p < 0.0001. (**B**) Representative IHC staining for PRDX2 in the invasive front reveals clustering of tumor cells with PRDX2 accumulation. (**C**) Invasive and migratory capacities of CD133^+^CD44^+^ CCSCs generated from HCT116/HT29 control or shPRDX2 cells (left panel). The bars represent the means ± SD of invaded/migrated cells from three independent experiments performed in duplicate (right panel). Statistical analysis: Student’s *t*-test, ^***^p < 0.001, ^****^p < 0.0001. (**D**) Adhesive capacity of HCT116/HT29-control- or -shPRDX2- CD133^+^CD44^+^ CCSCs to fibronectin and type 1 collagen, respectively. Percent adhesion was calculated as the number of adhesive cells/adhesive cells + nonadhesive cells. The data are presented as the percent of adhesive cells observed in three fields per assay and are expressed as an average of triplicate determinations. Statistical analysis: Student’s *t*-test, ^*^p < 0.05, and ^**^p < 0.01.

In a previous study, we provided evidence that PRDX2 expression serves as an independent and unfavorable prognostic indicator for stage I-III colorectal cancer patients [41]. However, little is known regarding the pattern of PRDX2 expression in stage IV patients. In the present study, we assessed PRDX2 expression in these patients by IHC, with 10 tumor tissues with liver metastasis collected from these individuals. The IHC results revealed that PRDX2 was highly expressed in colon cancer with liver metastasis (LM) compared to nonmetastatic tissues (NM) and matched normal colon tissues (MN) (Figure 4A). In addition, in the invasion front of tumor tissues, PRDX2 overexpression was also predominantly observed (Figure 4B). These results suggest that PRDX2 likely plays a role in tumor invasiveness. Next, based on the widely recognized view that CSCs are responsible for tumor metastasis, we assessed whether PRDX2 is functionally associated with the migration and invasion of CD133^+^CD44^+^ CSCs *in vitro*. The results revealed that migration and invasion was attenuated in PRDX2 knockdown CD133^+^CD44^+^ CCSCs (Figure 4C). Moreover, PRDX2 knockdown CD133^+^CD44^+^ CCSCs displayed lower adhesive capacity to fibronectin or type I collagen compared than the cells transfected with the scramble control (Figure 4D).

### PRDX2 knockdown inhibits the metastatic capacity of CCSCs

**Figure 5:**
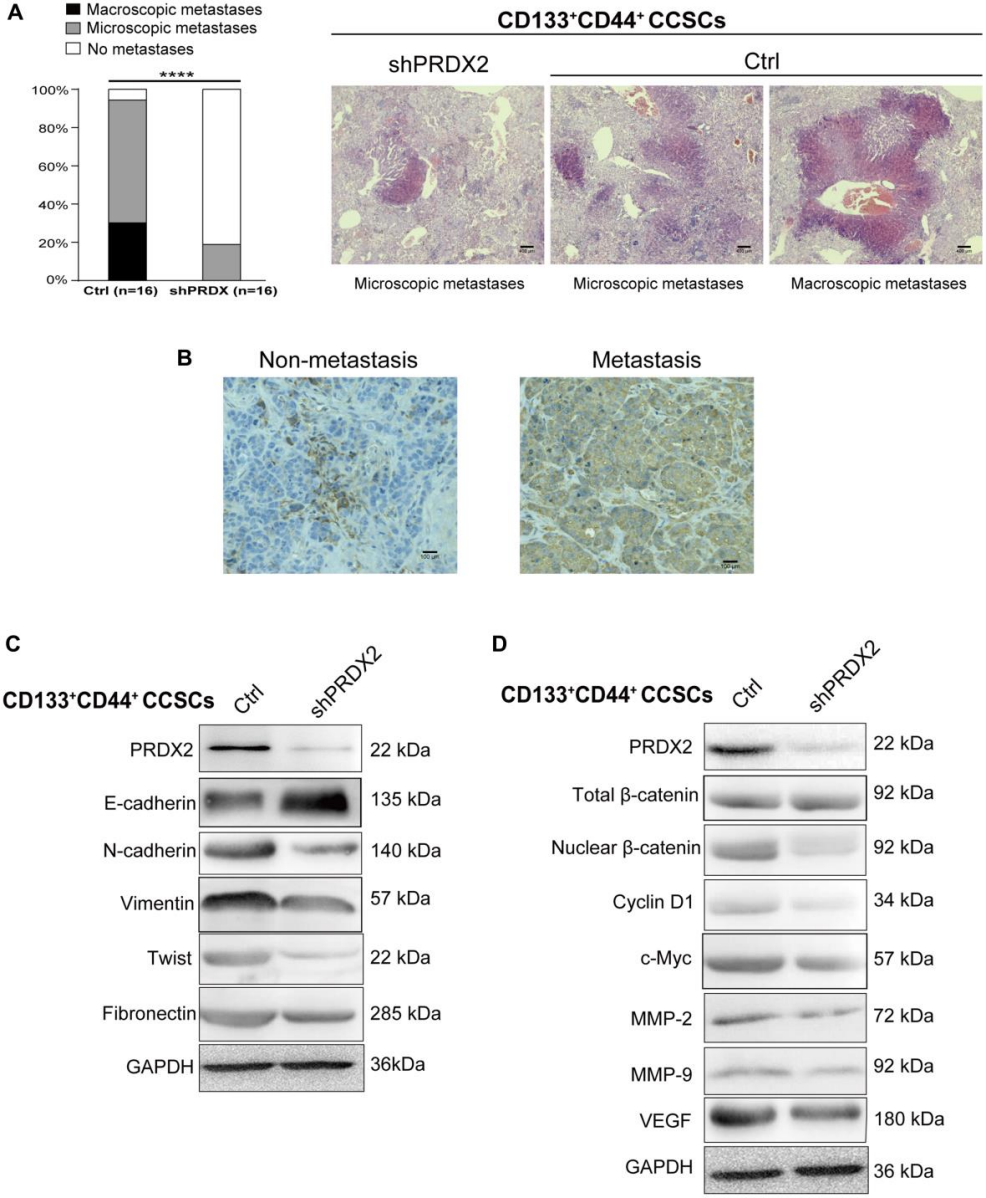
**PRDX2 knockdown inhibits the metastatic capacity of CCSCs.** (**A**) Histological analysis of the liver for metastatic lesions was performed by hematoxylin and eosin staining. The metastatic status of the mice (*n* = 16 per group) with orthotopic implantation of 1 × 10^4^ cells dissociated from HCT116/HT29-control or -shPRDX2 CD133^+^CD44^+^ CCSCs is provided separately as macroscopic (black) and microscopic (gray) evidence for metastasis (left panel). Statistical analysis: Fisher’s exact test, ^***^p < 0.0001. Representative images are from two mice receiving control-HCT116-CD133^+^CD44^+^ CCSCs and a mouse receiving shPRDX2-HCT116-CD133^-^CD44^-^ CCSCs (right panel). (**B**) Representative IHC staining for PRDX2 reveals that higher PRDX2 expression was detected in the orthotopic tumor tissues from metastatic cases than in tumor samples from nonmetastatic mice. (**C**) Western blot analysis of EMT protein expression in lysates of control- and shPRDX2-CD133^+^CD44^+^ CCSCs. (**D**) Western blot analysis of Wnt/β-catenin signaling pathway protein expression in lysates of control- and shPRDX2-CD133^+^CD44^+^ CCSCs.

The *in vitro* results described above prompted us to further investigate the effect of PRDX2 knockdown on the metastasis of CD133^+^CD44^+^ CCSCs *in vivo*. To this end, 1 × 10^4^ control- or shPRDX2-CD133^+^CD44^+^ CCSCs were injected into the cecal wall of 16 nude mice to establish a orthotopic tumor transplant models. After 14–18 weeks, macroscopic and microscopic analysis of the livers revealed that only mice injected with control CD133^+^CD44^+^ CCSCs developed macroscopic liver metastases, and the number of microscopic liver metastases was also significantly higher in mice injected with control CD133^+^CD44^+^ CCSCs than in those injected with shPRDX2 knockdown CD133^+^CD44^+^ CCSCs (Figure 5A). Moreover, a markedly decreased proportion of mice without any trace of liver metastases was observed in the mice injected with shPRDX2 knockdown CD133^+^CD44^+^ CCSCs (Figure 5A). In addition, the IHC results showed that PRDX2 expression was enhanced in the orthotopic tumor tissues from metastatic mice compared to tumor samples from nonmetastatic mice (Figure 5B). These data further confirmed the crucial role of PRDX2 in the metastasis of CD133^+^CD44^+^ CCSC tumors. Subsequently, we assessed whether the stem cell function-regulating role of PRDX2 in CD133^+^CD44^+^ CCSCs involves Wnt signaling and the EMT process which play crucial roles in colon cancer initiation, metastasis and stemness maintenance [42, 43]. In the EMT process, we observed that PRDX2 knockdown in CD133^+^CD44^+^ CCSCs led to a concomitant downregulation of EMT markers and the upregulation of E-cadherin (Figure 5C). Regarding, Wnt signaling, no significant change in total β-catenin expression was observed between the PRDX2-depleted CD133^+^CD44^+^ and control CCSCs. However, both intranuclear β-catenin expression and metastasis-related Wnt target gene downregulation was remarkedly decreased in PRDX2-depleted CD133^+^CD44^+^ CCSCs (Figure 5D).

### PRDX2 knockdown sensitizes CCSCs to chemotherapeutics

**Figure 6:**
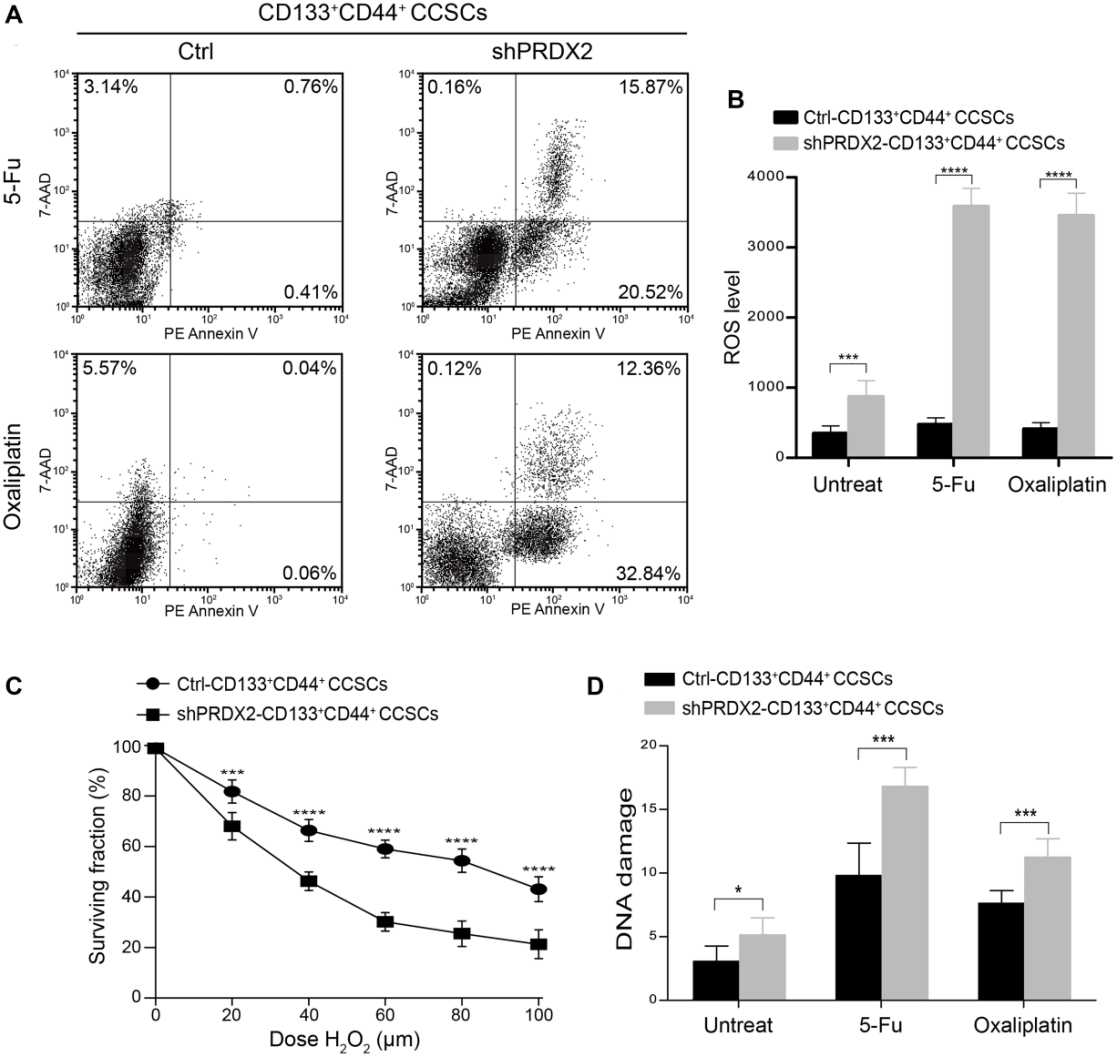
**PRDX2 knockdown sensitizes CCSCs to chemotherapeutics.** (**A**) Effect of 24 hours exposure of control- and shPRDX2-HT29-CD133^+^CD44^+^ CCSCs to 500 μg/mL of 5-FU or 100 μM oxaliplatin, as determined by flow cytometry apoptosis analysis with PE-labeled Annexin V containing 7-AAD staining. (**B**) Intracellular ROS levels of control- and shPRDX2-HT29-CD133^+^CD44^+^ CCSCs were measured before drug exposure and 24 hours after 500 μg/mL 5-FU or 100 μM oxaliplatin exposure. ROS levels are presented as the means ± SD of fluorescence intensity measured by flow cytometry with a DCFH-DA probe. Three independent experiments were performed in triplicate. Statistical analysis: Student’s *t*-test, ^***^p < 0.001, and ^****^p < 0.0001. (**C**) Survival curves obtained from clonogenic assays of control- and shPRDX2-HT29-CD133^+^CD44^+^ CCSCs exposed to different H_2_O_2_ doses. The data are presented as the means ± SD of three independent experiments performed in triplicate for each dose. Statistical analysis: Student’s *t*-test, ^***^p < 0.001, and ^****^p < 0.0001 vs. shPRDX2. (**D**) DNA damage of control- and shPRDX2-HT29-CD133^+^CD44^+^ CCSCs was measured before drug exposure and 24 hours after exposure to 500 μg/mL of 5-FU or 100 μM oxaliplatin by alkaline comet assay. The data are presented as the means of median tail moments ±SD of three independent experiments performed in triplicate. Statistical analysis: Student’s *t*-test, ^*^p < 0.05, and ^***^p < 0.001.

CSCs are well known to exhibit higher ROS levels than non-CSCs, which is thought to be an important cause of cancer chemoresistance [44, 45]. Thus, as PRDX2 is a crucial antioxidant gene, we were particularly interested in investigating the relationship between PRDX2 and CD133^+^CD44^+^ CCSC-induced chemoresistance. To this end, control- or shPRDX2-HCT29-CD133^+^CD44^+^ CCSCs were exposed to 500 μg/ml of 5-FU or 100 μM oxaliplatin for 24 h, and cell apoptosis was subsequently analyzed by flow cytometry with PE-labeled Annexin V containing 7-amino-actinomycin (7-AAD). In PRDX2 knockdown CD133^+^CD44^+^ CCSCs, some of these CCSCs undergoing apoptosis were in the latest stage of apoptosis after drug exposure (Figure 6A). In contrast, almost all of the control CCSCs were viable and did not undergo apoptosis, even when exposed to such high drug concentrations (Figure 6A). These findings further confirm the chemoresistance of CD133^+^CD44^+^ CCSCs and suggest that PRDX2 plays a crucial regulatory role in this process. Based on these findings, the regulatory mechanism of PRDX2 in the chemoresistance of CD133^+^CD44^+^ CCSCs was further evaluated. First, we assessed the endogenous production of ROS in control- and shPRDX2-CD133^+^CD44^+^ CCSCs with or without subsequent exposure to OXLP or 5-FU. In the absence of drug exposure, the intracellular ROS levels in PRDX2-depleted CD133^+^CD44^+^ CCSCs was more than twice as high as that observed in the control CCSCs, while a higher levels of intracellular ROS levels were also observed in PRDX2-depleted CD133^+^CD44^+^ CCSCs after exposure to OXLP or 5-FU, as determined by flow cytometry analysis (Figure 6B). Moreover, because PRDX2, as one of the most efficient intracellular H_2_O_2_ scavengers, can catalyze peroxide reduction to balance cellular hydrogen peroxide (H_2_O_2_) levels [27], we performed a clonogenic survival assays of control- and shPRDX2-CD133^+^CD44^+^ CCSCs following exposure to H_2_O_2_. Exogenous H_2_O_2_ treatment resulted in a dose-dependent reduction in cell survival (Figure 6C), which was more pronounced in PRDX2 knockdown CD133^+^CD44^+^ CCSCs than in control CCSCs (Figure 6C). Thus, these data indicate that PRDX2 knockdown in CD133^+^CD44^+^ CCSCs can sensitize these cells to oxidative stress. Because enhanced ROS levels in CSCs are consistent with increased DNA damage after exposure to chemotherapeutics [43, 46], DNA injury was evaluated through an alkaline comet assay. Our results showed that PRDX2 knockdown CD133^+^CD44^+^ CSCs displayed enhanced DNA strand breaks in both the absence or presence of OXLP or 5-FU exposure (Figure 6D).

## DISCUSSION

The CSC model describes cancers as being hierarchically organized, which is similar to normal stem tissues or cells [4]. According to this model, only a small fraction of cells, also called tumor-initiating cells or CSCs, have extensive self-renewal and differentiation abilities [8]. This cell subpopulation is currently a focus of cancer research, as it is thought to be a major factor in tumor initiation, metastasis, progression, drug resistance, and poor prognosis [18]. Thus, an effective treatment method for cancer should focus on CSCs, not only bulk tumor cells. Based on putative cell surface markers and their combination with stem cell-like features, several unique CSC populations have been identified in hematogenous tumors and most solid tumors [5, 9]. In particular, in colon cancer, CCSCs have been identified in established colon cancer cell lines or in primary tumors through their expression of CD133, CD44, CD166, Lgr5, EpCAM, ALDH1 and β-catenin alone or through combinations of these markers [10–17]. A previous study demonstrated that, in contrast to bulk tumor cells, rare CD133^+^CD44^+^ CCSCs are notably distinct at the genomic, morphological and functional levels, showing extensive self-renewal ability *in vivo* and *in vitro* and possessing the capacity to produce different cell phenotypes [23, 47]. Growing evidence has indicated that CD133 colocalizes in the same region as CD44, and the combination of CD133 and CD44 may be the most active CCSC population [22]. These results suggest that to eradicate colon cancer, CD133^+^CD44^+^ CCSCs should be specifically and effectively and targeted and eliminated by anticancer treatment.

Peroxiredoxins (PRDXs, including PRDX1-6) are a ubiquitous family of antioxidant enzymes known to catalyze peroxide reduction to balance the cellular levels of hydrogen peroxide (H_2_O_2_) [48, 49]. Peroxiredoxins are involved in a variety of signaling pathways, such as the NF-κB signaling, STAT3, Wnt/β-catenin and MAPK kinase pathways, which are closely associated with cancer development [35]. In particular, the tumor-promoting effect of PRDX2 is well established in several cancers [29, 32, 33, 50]. Notably, compared to the other PRDXs, PRDX2 was identified in our previous study as the most highly expressed upregulated protein in colorectal tumor tissue [51]. our previous study also demonstrated that PRDX2 knockdown inhibited the growth of colorectal cancer cells in part by downregulating Wnt/β-catenin signaling [29]. Subsequently, we also confirmed the expression of PRDX2 in colorectal cancer using a large number of colorectal cancer tissue samples (226 cases), and PRDX2 overexpression was identified an independent and unfavorable prognostic indicator in stage I-III, early stage (stage I-II) and advanced stage (stage III) colon cancer patients [41]. These results of these studies suggest that PRDX2 may have a tumor-promoting role in colon cancer. However, the importance of PRDX2 in CD133^+^CD44^+^ CCSCs is not fully clear. Here, we first investigated the relationship between PRDX2 and CD133/CD44 expression at the mRNA and protein levels. PRDX2 expression was remarkably higher and more frequently upregulated in CD133(+)/CD44(+) tumor tissues compared to CD133(-)/CD44(-) tumor tissues. A positive relationship between PRDX2 and CD133/CD44 expression at the transcriptional level was also observed. As sphere formation is a stem-like feature of CD133^+^CD44^+^ CCSCs, we further assessed PRDX2 expression in spheroid CD133^+^CD44^+^ CCSCs and adherent CD133^-^CD44^-^ cells. The results demonstrated that spheroid CD133^+^CD44^+^ CCSCs not only coexpressed CD133 and CD44 but also expressed high levels of PRDX2. In contrast, the adherent CD133^-^CD44^-^ cells showed minimal of CD133 and CD44 expression, and only scattered PRDX2 was detected in these cells. These results suggest that PRDX2 expression is closely associated with CD133^+^CD44^+^ CCSCs in colon cancer.

CCSCs are biologically different from differentiated tumor cells and are responsible for tumor initiation, metastasis and progression [6]. Thus, the therapeutic method to eradicate colon cancer should focus on eliminating CD133^+^CD44^+^ CCSCs. Several studies have investigated CSC interventions, which have produced some interesting results. For example, TCF4, a key factor in WNT signaling, was shown to bind LncCCAT1 to promote LncCCAT1 transcription, thereby enhancing the stemness, proliferation, and metastatic capacities of breast CSCs [52]. Moreover, Wang et al. reported that lnc-DILC can effectively inhibit liver CSC expansion by suppressing IL-6/STAT3 signaling [53]. In addition, SB-T-1214, a next-generation taxoid, not only inhibited stem cell spheroids induced by CD133^+^CD44^+^ CCSCs but also induced CD133^+^CD44^+^ CCSC apoptotic cell death [22]. Here, we investigated CD133^+^CD44^+^ CCSC-specific alterations induced by the important H_2_O_2_ scavenger PRDX2. Specifically, two highly invasive colon cancer cell lines, HCT116 and HT29, were evaluated in the present study, as a previous study reported that these cell lines harbor minority cell populations with the highest observed expression of CD133, which coincided with high CD44 expression [22]. Our results demonstrated that PRDX2 knockdown inhibits the self-renewal capacity of CD133+CD44+ CCSCs *in vitro* and the tumor incidence and growth of these cells *in vivo*. In addition, PRDX2 knockdown significantly decreased the invasiveness and liver metastatic potential of CD133^+^CD44^+^ CCSCs and sensitized these cells to chemotherapeutics. These results suggest that PRDX2 might play crucial roles in colon cancer progression by regulating CD133^+^CD44^+^ CCSC functions.

Wnt/β-catenin signaling is essential for normal and cancer stem cell homeostasis, and the deregulation of Wnt signaling is involved in malignant tumor behavior and cancer development [54]. Moreover, on the basis of the “β-catenin paradox”, nuclear β-catenin is highly localized and expressed in the colon cell stem cell niche and at the invasive tumor front, and enhanced Wnt signaling has been confirmed to induce EMT, suggesting a role for Wnt-induced CSCs in the propagation of colon cancer metastasis [55]. In the present study, we specifically investigated whether the crucial role of PRDX2 in CCSCs is involved in Wnt signaling and EMT. Our results demonstrated that PRDX2 silencing in CD133^+^CD44^+^ CCSCs did not change the total expression levels of β-catenin but decreased intranuclear β-catenin expression concomitant with the downregulation of metastasis-related Wnt target genes. Regarding the EMT process, we observed that PRDX2 knockdown in CD133^+^CD44^+^ CSCs led to a concomitant downregulation of EMT markers and an upregulation of E-cadherin. The EMT program in colon cancer has been shown to reflect tumor metastasis and is likely to account for these metastasis-related phenotypes, including tumor budding, generation of CSCs, circulating tumor cells, and drug resistance [56, 57]. In general, CSCs are naturally resistant to anticancer chemotherapy and radiotherapy, with the associated mechanisms involving higher ROS levels, lower DNA damage, resistance to apoptosis, and a profound capacity for DNA repair [58]. Therefore, the existence of CSCs has important implications for chemotherapy. PRDX2 is an important member of the ROS scavenging system, and the regulatory mechanism of PRDX2 in the chemoresistance of CD133^+^CD44^+^ CCSCs was assessed. Our results showed that PRDX2 knockdown sensitized CD133^+^CD44^+^ CCSCs to 5-FU and oxaliplatin, and the mechanisms associated with this effect included enhanced ROS production, a greater sensitivity to oxidative stress and increased DNA breakage in CD133^+^CD44^+^ CCSCs derived from PRDX2 knockdown cells.

In summary, we elucidated the crucial role of PRDX2 in CD133^+^CD44^+^ CCSCs and the underlying mechanisms. PRDX2 is highly expressed in CD133/CD44-positive colon cancer tissues and spheroid CD133^+^CD44^+^ CCSCs. The high expression of PRDX2 was closely associated with CD133^+^CD44^+^ CCSCs in colon cancer. We further showed that PRDX2 is functionally required for CD133^+^CD44^+^ CCSC stemness maintenance, tumor initiation, migration and invasion, and liver metastasis, and the expression of various EMT markers and Wnt/β-catenin signaling proteins was altered after PRDX2 inhibition. Additionally, PRDX2 knockdown led to an increased production of ROS in CD133^+^CD44^+^ CCSCs, sensitizing CCSCs to oxidative stress and chemotherapy. Importantly, the crucial stem cell function-inducing role of PRDX2 in CD133^+^CD44^+^ CCSCs may have important clinical implications for eradicating colon cancer.

## MATERIALS AND METHODS

### Cell culture

Authenticated human colon cancer cell lines HT29 and HCT116 were purchased from the Cell Bank of Type Culture Collection (Shanghai, China). All lines were maintained in McCoy’s 5a medium (Gibco, USA) supplemented with 10% fetal bovine serum (FBS) at 37°C under an atmosphere with 5% CO_2_.

### PRDX2 silencing in CD133^+^CD44^+^ CCSCs

To silence PRDX2 expression in CD133^+^CD44^+^ CCSCs, we first generated HCT116 and HT29 cells exhibiting stable PRDX2 knockdown (shPRDX2) via lentiviral vector-mediated specific shRNA delivery, and a nontarget negative control lentivirus vector was also transduced into these cells to control for the impact of the lentiviral vector. The lentiviral cloning vectors Ubi-shPRDX2-EGFP-Puromycin (LV-shPRDX2) (Sequence: TCTTTATCATCGATGGCAA) and Ubi-NC-EGFP-Puromycin (LV-control) (Sequence: TTCTCCGAACGTGTCACGT) were purchased from Genechem Co., Ltd. (Shanghai, China). The cells were subcultured in medium containing puromycin (10 μg/ml, Sigma-Aldrich, USA), and antibiotic-resistant clones were picked and passaged as stable cells. Next, these stably transfected cells (shPRDX2-HCT116, shPRDX2-HT29, control-HCT116, and control-HT29) were used to isolate shPRDX2-HCT116-CD133^+^CD44^+^ CCSCs, shPRDX2-HT29-CD133^+^CD44^+^ CCSCs, control-HCT116-CD133^+^CD44^+^ CCSCs, and control-HT29-CD133^+^CD44^+^ CCSCs by magnetic bead sorting. The use of this approach resulted in a considerable enrichment of CD133^+^CD44^+^ CCSCs (regular purity > 90%), as identified via flow cytometry analysis with a PE-labeled anti-CD133 antibody (Miltenyi Biotech, Germany) and a PE-cy5-labeled anti-CD44 antibody (eBiosciences, USA). Three independent experiments were performed.

### Immunohistochemistry analysis of tissue samples

Formaldehyde-fixed, paraffin-embedded sections were assessed by H&E staining and immunohistochemistry analysis following routine protocols as previously described [40]. Primary antibodies against PRDX2 (Abcam, USA), CD133 (Proteintech, USA), and CD44 (CST, USA) were used for immunohistochemistry.

### Quantitative reverse transcription RT-PCR

Quantitative reverse transcription PCR (RT-qPCR) was performed using SYBR Premix Ex Taq II (TaKaRa, Japan) and a C1000 Touch^TM^ Thermal Cycler and CFX Real-Time PCR Detection System (Bio-Rad, USA). The sequences of primers used for RT-qPCR were as follows: CD133 (forward 5′-ACA ATC CTG TTA TGA CAA GCC CA-3′; reverse 5′-GGA AAG TCC TTG TAG ACC CAG AAA-3′), PRDX2 (forward 5′-CAC CTG GCT TGG ATC AAC ACC-3′; reverse 5′-CAG CAC GCC GTA ATC CTC AG-3′), CD44 (forward 5′-ATC ATC TTG GCA TCC CTC TTG-3′; reverse 5′-CAC CAT TTC CTG AGA CTT GCT G-3′), and GAPDH (forward 5′-ACC ACA GTC CAT GCC ATC CAC-3′; reverse 5′-TCC ACC ACC CTG TTG CTG TA-3′).

### Immunofluorescence assay

Immunofluorescence analyses of spheroid CD133^+^CD44^+^ CCSCs and adherent CD133^-^CD44^-^ cells were performed following routine protocols as previously described [40] using rabbit polyclonal antibodies specific for PRDX2 (1:100), and CD44 (1:100) and mouse polyclonal antibodies specific for CD133 (1:100).

### Western blot analysis

Western blot analysis was performed using specific primary antibodies against CD133 (Proteintech, USA), CD44 (CST, USA), Lgr5 (Abcam, USA), EpCAM (Abcam, USA), ALDH1 (Abcam, USA), β-catenin (Proteintech, USA), Nanog (CST, USA), E-cadherin (CST, USA), N-cadherin (Epitomics, USA), vimentin (CST, USA), Snail (Abcam, USA), Twist (Abcam, USA), Slug (Abcam, USA), ZEB1 (Abcam, USA), fibronectin (Abcam, USA), Sox2 (CST, USA), Oct4 (CST, USA), PRDX2 (Abcam, USA), Cyclin D1 (Abcam, USA), c-Myc (Abcam, USA), MMP-2 (Abcam, USA), MMP-9 (Abcam, USA), VEGF (Abcam, USA) and GAPDH (Goodhere, China).

### Determination of apoptosis

The chemoresistance of control-HT29-CD133^+^CD44^+^ and shPRDX2-HT29-CD133^+^CD44^+^ CCSCs was evaluated by flow cytometry apoptosis analysis. Briefly, control-HT29-CD133^+^CD44^+^ and shPRDX2-HT29-CD133^+^CD44^+^ CCSCs were treated for 24 h with 500 μg/ml of 5-FU or 100 μM oxaliplatin. After 24 h of drug exposure, the cells were washed with cold PBS twice, resuspended in binding buffer and then adjusted to a density of 10^6^ cells/ml. Subsequently, 10^5^ cells were removed and mixed with 5 μl of Annexin V-PE and 5 μl of 7-AAD (BD Biosciences, USA) by gentle vortexing before being incubated for 15 min at RT in the dark. Finally, 400 μl of binding buffer was added to each tube, and cell apoptosis was analyzed by flow cytometry.

### Statistical analysis

The data are presented as the means ± standard deviation. Statistical analyses were performed using the unpaired two tailed Student’s *t*-test, paired *t*-test, and one-way ANOVA with Newman Keuls as post hoc test with GraphPad PRISM (San Diego, CA, USA). Differences were considered significant at *P* < 0.05 [^*^, *p* < 0.05; ^**^, *p* < 0.01; ^***^, *p* < 0.001; ^****^, *p* < 0.0001; and n.s., no significant difference (*p* ≥ 0.05)].

## Supplementary Materials

Supplementary Materials and Methods

Supplementary Figure 1
