# Supplementary material for: The critical role of peroxiredoxin-2 in colon cancer stem cells
Source: Aging (Albany NY). 2021 Mar 26;13(8):11170–87. doi: 10.18632/aging.202784 (PMC8109100; doi:10.18632/aging.202784)
Supplement: Supplementary Materials and Methods [file aging-13-202784-s001.pdf]

## SUPPLEMENTARY MATERIALS AND METHODS

### Isolation and identification of CD133$^+$CD44$^+$ CCSCs

Putative CD133$^+$CD44$^+$ CCSCs were isolated from cell populations by magnetic bead sorting using a magnetic activated cell sorting (MACS) MicroBead kit (Miltenyi Biotech, Germany). The cells were trypsinized, washed, and resuspended in PBS to prepare single-cell suspensions, which were then incubated with CD133 MicroBeads (Miltenyi Biotech, USA) for 20 min at 2-8°C, after which CD133$^+$ and CD133$^-$ cells were successfully separated using a MACS magnet and MS columns (Miltenyi Biotech, USA). Then, the isolated CD133$^+$ cells were incubated with CD44 MicroBeads (Miltenyi Biotech, USA), which was followed by cleavage of the MicroBeads until CD133$^+$CD44$^+$ cells were enriched. For CD133$^-$CD44$^-$ cell isolation, CD133$^-$ cells were used for depletion with a CD44 antibody. Purity assessments for positive and negative separation was performed by flow cytometry after magnetic bead separation. To this end, the samples were then stained with a phycoerythrin (PE)-labeled anti-CD133 antibody (Miltenyi Biotech, Germany) and a fluorescein isothiocyanate (FITC)-labeled anti-CD44 antibody (eBiosciences, USA) and analyzed using a FACSCalibur flow cytometer (BD Biosciences, USA). A mouse IgG1κ antibody conjugated to PE (Miltenyi Biotech, Germany) and a rat IgG2b κ antibody conjugated to FITC (eBiosciences, USA) were used as isotype controls. Three independent experiments were performed.

### Sphere formation assay

Isolated control- and shPRDX2-CD133$^+$CD44$^+$ CCSCs ($1 \times 10^4$ cells/well) were plated into 6-well Ultra Low Cluster plates (Corning, USA) and cultured in serum-free DMEM-F12 medium (Gibco, USA) supplemented with 20 ng/ml EGF (Peprotech, USA), B27 supplement (1:50, Gibco, USA) and 20 ng/ml basic FGF (Peprotech, USA). The number of cell spheres was counted after three weeks of cultivation.

### Animal studies

All animal protocols followed the Institutional Animal Ethics Care of Chongqing Medical University. To generate subcutaneous tumors, single-cell suspensions with >90% survival rates were subcutaneously injected into the lateral wall of 4-week-old female BALB/c nude mice. Tumor growth was observed and recorded every 1 week using the following formula: Volume = Width$^2$ × Length/2. Subcutaneous tumors were analyzed by histological or flow cytometry analysis. For the orthotopic implantation of tumor cells, mice were anesthetized, and the cecum was exteriorized by laparotomy. Then, control- and shPRDX2-CD133$^+$CD44$^+$ CCSCs suspended in 30 µl of DMEM F-12 medium and 20 µl Matrigel were injected into the cecal wall.

### Transwell migration and invasion assays

For migration assays, isolated control- and shPRDX2-CD133$^+$CD44$^+$ CCSCs were resuspended to a density of $1 \times 10^4$ cells in 250 µl of DMEM-F12 and placed in the upper chamber of transwells with the noncoated membrane (Millipore, USA). Medium supplemented with growth factors was used as a chemoattractant in the lower transwell chamber. After 24 h, the cells were fixed with 4% paraformaldehyde for 30 min, stained for 10 min in 0.5% crystal violet, and then counted under a light microscope at 100× magnification. The invasion assays were performed similarly to the migration assays except that Matrigel-coated membrane was used in the top chamber (Millipore, USA).

### Analysis of CCSC adhesion to ECM proteins

Isolated control- and shPRDX2-CD133$^+$CD44$^+$ CCSCs were plated into 60-mm dishes ($5 \times 10^5$ cells per dish) coated with type I collagen or fibronectin, respectively, and cultured for 12 h. Nonadhesive and adhesive cells were then collected and counted using a hemocytometer.

### Analysis of intracellular ROS levels

Intracellular ROS levels of control- and shPRDX2-HT29-CD133$^+$CD44$^+$ CCSCs were measured before drug exposure and 24 hours after 500 µg/mL 5-FU or 100 µM oxaliplatin exposure. ROS were detected with a 2,7-dichlorofluorescein diacetate (DCFH-DA) probe (Beyotime, China) according to the manufacturer’s instructions. Briefly, the cells were incubated with 10 µmol/L DCFH-DA at 37°C for 20 min, after which they were washed 3 times with PBS to remove excess DCFH-DA, suspended in medium and then immediately analyzed by flow cytometry.

### Alkaline comet assay of DNA damage

DNA damage was evaluated using an alkaline comet assay following a previously described procedure with some modifications [1]. Briefly, single-cell suspensions of control- and shPRDX2-HT29-CD133$^+$CD44$^+$ CCSCs with or without exposure to chemotherapy drugs were prepared. These cells were then embedded in low-melting-point agarose and lysed overnight at 4°C in

lysis buffer (0.1 M EDTA, 1% Triton X-100, 0.01 M Tris base, 2.5 M NaCl, and 5% DMSO, pH 10). Then, the unwinding step was performed in unwinding/electrophoresis buffer (0.3 M NaOH, 2 mM EDTA, pH 13) for 1 h at 4°C. Electrophoresis was conducted for 25 min at 4°C in unwinding/electrophoresis buffer at electric-field strength of 306 mA and 0.6 V/cm. The slides were then neutralized with a neutralization buffer (0.4 Tris-HCl, pH 7.5), rinsed with distilled water, air-dried, and then stained with 20 µl ethidium bromide (2 µg/mL) before being covered with standard cover slips. Subsequently, the slides were analyzed for comets with a Nikon Optiphot microscope attached to a Pulnix video camera and fluorescence image analysis system. DNA single-strand breaks were determined using the tail moment parameter.

## REFERENCE


1. Singh NP, McCoy MT, Tice RR, Schneider EL. A simple technique for quantitation of low levels of DNA damage in individual cells. Exp Cell Res. 1988; 175:184–91. https://doi.org/10.1016/0014-4827(88)90265-0 PMID:3345800
