# Supplementary material for: The critical role of peroxiredoxin-2 in colon cancer stem cells
Source: Aging (Albany NY). 2021 Mar 26;13(8):11170–87. doi: 10.18632/aging.202784 (PMC8109100; doi:10.18632/aging.202784)
Supplement: Supplementary Figure 1 [file aging-13-202784-s002.pdf]

SUPPLEMENTARY FIGURE

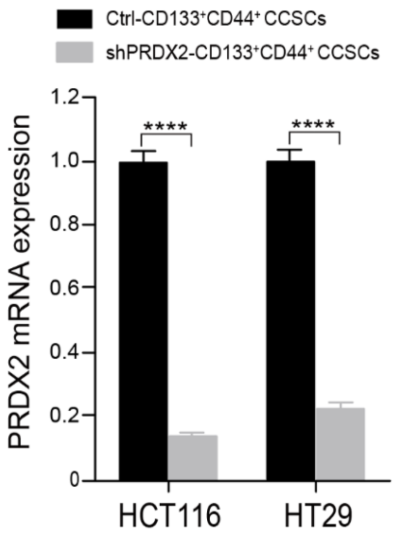

Supplementary Figure 1. The mRNA levels of PRDX2 in CD133+CD44+ CCSCs generated from HCT116/HT29 control or shPRDX2 cells were detected by quantitative polymerase chain reaction. GAPDH is used as the loading controls. The data are the mean ± SD of spheres number of three independent experiments with triplicates. Statistical analysis: Student's *t*-test, $^{****}p < 0.0001$.
